# Phenotypic drug susceptibility characterization and clinical outcomes of tuberculosis strains with A-probe mutation by GeneXpert MTB/RIF

**DOI:** 10.1186/s12879-023-08509-0

**Published:** 2023-11-27

**Authors:** Qi Nie, Dan Sun, Muxin Zhu, Shengjin Tu, Nanshan Chen, Hua Chen, Yong Zhou, Ge Yao, Xiaoqing Zhang, Tongcun Zhang, Chengfeng Yang, Lixuan Tao

**Affiliations:** 1 College of Life Sciences and Health, Wuhan University of Science and Technology, Hubei, China; 2 Department of MDR/RR-TB, Wuhan Jinyintan Hospital, Tongji Medical College of Huazhong, Wuhan Research Center for Communicable Disease Diagnosis and Treatment, University of Science and Technology, Hubei Clinical Research Center for Infectious Diseases, Chinese Academy of Medical Sciences, Hubei, China; 3 Department of Interventional therapy, Wuhan Pulmonary Hospital, Hubei, China; 4 Hubei Provincial Center for Disease Control and Prevention, Hubei, China; 5 Emergency Department, Puren Hospital, Wuhan University of science and technology, Hubei, China

**Keywords:** Mycobacterium tuberculosis, Mutation, Rifampicin-resistant, GeneXpert MTB/RIF, Clinical outcome

## Abstract

**Background:**

GeneXpert MTB/RIF (Xpert) assay was applied widely to detect Mycobacterium tuberculosis (MTB) and rifampicin resistance.

**Methods:**

Retrospectively investigated the association among treatment histories, phenotypic drug susceptibility testing (pDST) results, and clinical outcomes of patients infected with probe A absent mutation isolate confirmed by Xpert.

**Results:**

63 patients with only probe A absent mutation and 40 with additional pDST results were analyzed. 24 (60.0%) patients had molecular-phenotypic discordant rifampicin (RIF) susceptibility testing results, including 12 (12/13, 92.3%) new tuberculosis (TB) patients and 12 (12/27, 44.4%) retreated ones. 28 (28/39, 71.8%) retreated patients received first-line treatment regime within two years with failed outcomes. New patients had better treatment outcomes than retreated ones (successful: 83.3% VS. 53.8%; *P* value = 0.02). The clinical results of RIF-susceptible TB confirmed by pDST were not better than RIF-resistant TB (successful: 62.5% VS. 50.0%; *P* value = 0.43). INH-resistant TB and INH-susceptible TB had similar treatment outcomes too (successful: 61.5% VS. 50.0%; *P* value = 0.48). 11 (11/12, 91.7%) new patients treated with the short treatment regimen (STR) had successful outcomes.

**Conclusions:**

More than half of mono probe A absent isolates had RIF molecular-phenotypic discordance results, especially in new patients. Probe A mutations were significantly associated with unsuccessful clinical outcomes, whether the pDST results were RIF susceptible or not. STR was the best choice for new patients.

**Trial registration:**

retrospectively registered in Wuhan Jinyintan Hospital (No. 2021-KY-16).

## Background

Drug-resistant tuberculosis (DR-TB) is a global health problem of great concern. Especially multidrug-/rifampicin-resistant tuberculosis (MDR/RR-TB) poses a great threat to TB control programs worldwide. Rapid and accurate detection of mycobacterium tuberculosis (MTB) and drug resistance is crucial for the appropriate treatment of patients suffering from tuberculosis (TB) and the prevention of spread of DR-TB. In some instances, the diagnosis of DR-TB is difficult and time-consuming, slowed down by the time it takes for a culture to become positive and the turnover time for drug susceptibility testing (DST), which may take approximately four to six weeks [1]. In addition, phenotypic DST (pDST) is prone to a high contamination rate, and the accuracy of susceptibility testing results varies with the tested drug and the DST method [2, 3].

Rifampicin (RIF) blocks RNA transcription in MTB by binding to the β-subunit (encoded by the *rpoB* gene) of the DNA-dependent RNA polymerase [4]. More than 95% RIF resistance is associated with mutations within an 81-bp RIF resistance-determining region (RRDR) of the *rpoB* gene, which comprises codons 507 to 533 [5]. The GeneXpert MTB/RIF (Xpert; Cepheid Xpert Inc., Sunnyvale, CA, USA) is a fully automated ultra-sensitive hemi-nested real time (RT)-PCR-based assay, which allows the simultaneous detection of the MTB and RIF resistance-conferring mutations using five molecular beacon overlapping probes by identifying the RRDR region, including disputed mutations it may carry, directly on clinical specimens [6, 7]. These probes are named probe A (codons 507–511), probe B (codons 512–518), probe C (codons 518–523), probe D (codons 523–529), and probe E (codons 529–533) [8]. This molecular assay reduces the time for Mycobacterium tuberculosis (MTB) and RIF resistance detection to within 2 h [9].

Despite the Xpert assay being highly sensitive and specific for RIF resistance, many uncertainties remain [10]. The high-confidence *rpoB* gene mutations that encode D516V, H526Y, H526D, and S531L (Escherichia coli numbering) are associated with RIF resistance in MTB [11, 12]. In contrast, L511P, M515V, D516Y, H526C, H526L, H526N, H526S, L533P, and I572F are disputed *rpoB* mutations with discordant pDST results [13–15]. In our previous research on shorter treatment regimens for MDR/RR-TB, we reported that five RR-TB strains with probe A mutations showed phenotypic drug sensitivity [16]. A retrospective study was conducted to evaluate the incidence of discordant pDST results in probe A mutation isolates, treatment outcomes, and the impact of treatment history on them.

## Materials and methods

### Study design and patient classification

This retrospective study evaluated a cohort of MDR/RR-TB patients from December 2016 to May 2021, whose specimens had mono probe A mutations determined by Xpert assay in the Department of Microbiology Laboratory, Wuhan Jinyintan hospital, Hubei, China. The new cases were patients who had never been treated for TB or had taken anti-TB medicines for less than one month. The retreated patients belonged to three groups, according to their previous anti-tuberculosis treatment history. Retreatment 1 group (R1): received only first-line drugs within two years. Retreatment 2 group (R2): Received only first-line drugs two years ago. Retreatment 3 group (R3): received second-line drugs in the past.

### Gene Xpert MTB/RIF assay

The Xpert assay involved the following manual steps according to the manufacturer’s instructions: sample reagent was added to specimens in a 2:1 ratio, inverted the samples twice during 15-mins incubation period at room temperature, transferred 2mL of liquefied sputum into the cartridge, loading of the cartridge into the device for the assay, and viewed the results in the Xpert software. The reports with mono probe A mutations provided by the GeneXpert software were analyzed.

### Mycobacterium tuberculosis culture and pDST

MTB culture was performed by the BACTEC Mycobacterial Growth Indicator Tube (MGIT) 960 system (BD, Sparks, MD, USA) as recommended, and used the MYCOTB MIC plate to test pDST according to the manufacturer’s instructions.

### Anti-tuberculosis treatment regimens

(1) First-line treatment regimen (FTR): the regimen was composed of four drugs from first-line, such as isoniazid (INH, H), rifampicin (RIF, R), pyrazinamide (PZA, Z), ethambutol (EMB, E), and streptomycin (SM, S). Levofloxacin (LFX) could replace one of above first-line drugs due to intolerance. The course of treatment was 6–12 months. (2) Short-term treatment regimen (STR): the regimen included at least two or more second-line anti-tuberculosis drugs, with a course of treatment not exceeding 12 months. The category included fluoroquinolone (FQ, including GFX/MFX/LFX, GFX = gatifloxacin, MFX = moxifloxacin)- and amikacin (AM)/linezolid (LZD)- based STR (4–6 FQ-AM/LZD-CFZ-PTO/CS-H-Z-E/5–6 FQ-CFZ-Z-E, CFZ = clofazimine, PTO = Prothionamide, CS = cycloserine) [16], FQ- and bedaquoline (BDQ)- based all-oral STR (6 BDQ-FQ-CFZ/LZD-PTO/CS-H-Z-E/5–6 FQ-CFZ-Z-E), and 3 LFX-AM-H-Z-E/9 LFX-H-Z-E [3]. Long-term treatment regimen (LTR): 18–24 months therapeutic schedule according to World Health Organization (WHO) guidelines [17–19].

### Definitions of clinical outcome and drug-resistant tuberculosis

#### Definitions of clinical outcome

Determination of the end of treatment outcome followed WHO outcome categories - Definitions and reporting framework for tuberculosis − 2013 revision (updated December 2014 and January 2020) - for MDR-TB programs (WHO, 2013). Cured or treatment completed belonged to successful outcomes, whereas unsuccessful or unfavorable ones included lost to follow-up (LTFU), death, failure, and relapse.

#### Definitions of drug-resistant tuberculosis [19, 20]

Hr-TB: TB caused by MTB strains resistant to INH and susceptible to RIF. MDR-TB: TB caused by MTB strains resistant to at least both RIF and INH. RR-TB: TB caused by MTB strains resistant to RIF. MDR-TB and RR-TB cases are often grouped as MDR/RR-TB and are eligible for treatment with MDR-TB regimens. Extensively drug-resistant tuberculosis (XDR-TB): TB fulfill the definition of MDR/RR-TB and which are also resistant to any FQ and at least one additional Group A drug (Group A drug including FQ, BDQ, and LZD). pre-XDR-TB: TB fulfill the definition of MDR/RR-TB and which are also resistant to any FQ.

### Data collection and statistical analysis

Demographic data were abstracted from Wuhan Jinyintan hospital information system (HIS). The following variables were abstracted from medical records: sex, age, address, patient categories, complication, sample source, pDST, medical history, treatment course and outcome data. Data were collected in an excel sheet and all results were expressed in percentages. Statistical analysis was performed using SPSS, version 25.0 (SPSS Inc., Chicago, USA), and a *p*-value less than 0.05 was considered statistically significant.

## Results

### Cases screening (Fig. 1)

**Fig. 1:**
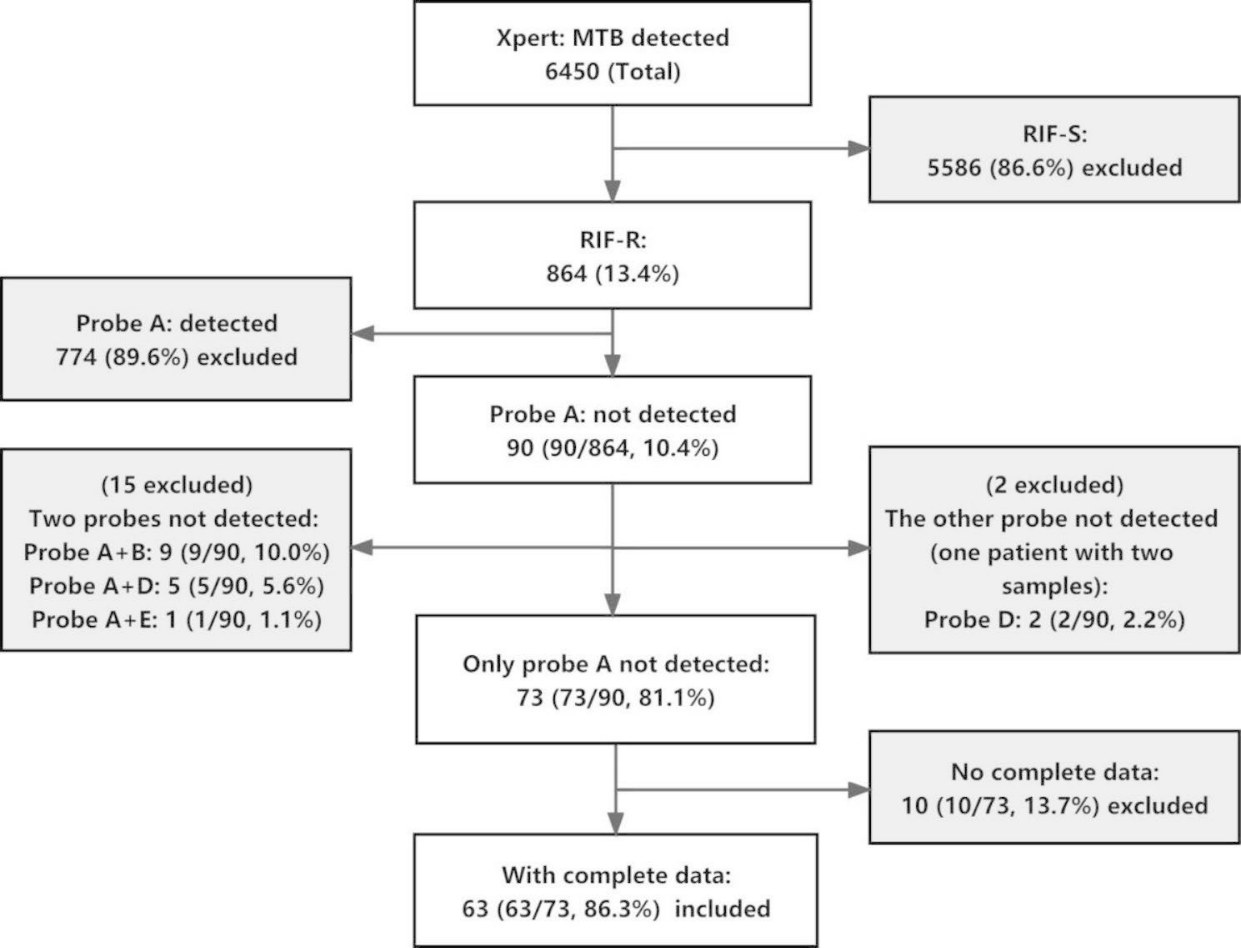
Outline of steps to identify the final research objectives. Xpert: GeneXpert MTB/RIF; MTB: Mycobacterium tuberculosis; RIF-S: rifampicin susceptibility; RIF-R: rifampicin resistance

Between December 2016 and May 2021, 6450 MTB isolates from different patients were identified by Xpert assay, and 864 (13.4%) were RIF-resistant. Of these, 90 (10.4%) isolates had mutations with absent probe A. Finally, 63 (1.0%) patients with mono probe A absence and complete medical records, were included in the analysis.

### Characteristics of cases

We analyzed demographic characteristics, treatment histories of TB, pDST results, and clinical outcomes. Among all 63 patients, 44 (69.8%) were males; 24 (38.1%) were new and 39 (61.9%) were retreated cases. The mean age was 37.5 ± 18.5 years (range 15–75 years), and six patients were under 18 years old, including two 15-year-old patients, three 16-year-old patients, and one 17-year-old patient. 7 (11.1%) with type 2 diabetes mellitus, 1 (1.6%) with human immunodeficiency virus (HIV) co-infection, 1 (1.6%) with nontuberculous mycobacteria (NTM) co-infection, and 3 (4.8%) with chronic hepatitis B. The majority (53, 84.1%) of the specimens were sputum or bronchoalveolar lavage fluid (BALF), while the remaining 10 (15.9%) were non-respiratory samples, including pleural effusion/empyema/tissue (3, 30.0%), lymph nodes (3, 30.0%), abdominal abscess (2, 20.0%), epididymal abscess (1, 10.0%), and paravertebral abscess (1, 10.0%). Except that RIF-susceptible tuberculosis (RS-TB, confirmed by pDST) rate in new patients was higher than in retreated patients (12/13, 92.3% VS. 12/27, 44.4%; *P* value < 0.05), there was no statistical difference for isoniazid or rifampicin pDST results detected between different groups (for example: male and female, >60 years and ≤60 years, with and without complications, respiratory and non-respiratory specimens; *P* value>0.05).

### TB treatment history, phenotypic DST results, and treatment outcomes

#### TB treatment history and pDST results (tables 1 and 2)

**Table 1:** Characteristics and phenotypic DST of cases included in the study

Patient characteristic	Total (N = 63, %)	Phenotypic DST (N’=40)					
		INH-S (%)	INH-R (%)	*P* value	RIF-S (%)	RIF-R (%)	*P* value
**Sex**							
Male	44(69.8)	12(85.7)	18(69.2)	0.44	19(79.2)	11(68.8)	0.71
Female	19(30.2)	2(14.3)	8(30.8)		5(20.8)	5(31.3)	
**Age category (year)**							
≤ 60	54(85.7)	12(85.7)	21(80.8)	0.97	21(87.5)	12(75.0)	0.55
> 60	9(14.3)	2(14.3)	5(19.2)		3(12.5)	4(25.0)	
**Complication**							
No complications	53(84.1)	9(64.3)	24(92.3)	0.07	20(83.3)	13(81.3)	0.80
With complications^#^	10(15.9)	5(35.7)	2(7.7)		4(16.7)	3(18.8)	
**Specimen type**							
Respiratory specimens	53(84.1)	13(92.9)	23(88.5)	0.91	23(95.8)	13(81.3)	0.33
Non-respiratory specimens^*^	10(15.9)	1(7.1)	3(11.5)		1(4.2)	3(18.8)	
**TB treatment history**							
New	24(38.1)	6(42.9)	7(26.9)	0.50	12(50.0)	1(6.3)^&^	0.00
Retreatment	39(61.9)	8(57.1)	19(73.1)		12(50.0)	15(93.8)	

^#^: included HIV infection, or diabetes mellitus, or hepatitis B, or nontuberculous mycobacteria (NTM) co-infection; ^*^:included pleural cavity (pleural tissue, pleural effusion), or abdominal cavity (perihepatic abscess, abdominal dropsy), or epididymal abscess, or paravertebral abscess, or lymphaden; ^&^: came from Singapore, the only overseas imported case; INH: isoniazid; RIF:rifampicin; R: resistant; S: susceptible; TB: Tuberculosis; DST: drug susceptibility test; New: patients who had never received treatment for TB or had taken anti-TB drugs for < 1 month.

**Table 2:** History of TB treatment and Phenotypic DST

Phenotypic DST (N = 40)	History of TB treatment				Total (%)
	New (%)	R1 (%)	R2 (%)	R3 (%)	
**RS-TB**	**12(92.3)**	**7(43.8)**	**3(50.0)**	**2(40.0)**	**24(60.0)**
Hs	6(46.2)	5(31.3)	1(16.7)	1(20.0)	13(32.5)
Hr	6(46.2)	2(12.5)	2(33.3)	1(20.0)	11(27.5)
**RR-TB**	**1(7.7)**	**9(56.3)**	**3(50.0)**	**3(60.0)**	**16(40.0)**
RMR/RPR	0(0.0)	1(6.3)	0(0.0)	0(0.0)	1(2.5)
MDR	1(7.7)	7(43.8)	1(16.7)	0(0.0)	9(22.5)
XDR/pre-XDR	0(0.0)	1(6.3)	2(33.3)	3(60.0)	6(15.0)
**Total**	**13(32.5)**	**16(40.0)**	**6(15.0)**	**5(12.5)**	**40(100.0)**

TB: tuberculosis; DST: drug susceptibility test; RS-TB: rifampicin-susceptible tuberculosis; RR-TB: rifampicin-resistant tuberculosis; Hs: isoniazid-susceptible; Hr: isoniazid-resistant; RMR/RPR: rifampicin mono- or poly- drug resistant; MDR: multidrug-resistant; XDR/pre-XDR: extensively drug resistant or pre-extensively drug resistant; New: patients who had never received treatment for TB or had taken anti-TB drugs for < 1 month; R1: retreatment 1 group, received only first-line drugs within two years. R2: retreatment 2 group, received only first-line drugs two years ago. R3: retreatment 3 group, received second-line drugs in the past.

Forty samples from 13 new and 27 retreated patients had pDST results. 24 (24/40, 60.0%) pDST results that from 12 (12/13, 92.3%) new patients (except the only one overseas imported case was MDR-TB) and 12 (12/27, 44.4%) retreated patients had molecular-phenotypic discordant RIF susceptibility results, including 2 (2/24, 8.3) FQ-resistant isolates, 6 (6/24, 25.0%) INH mono-resistant isolates, 1 (1/24, 4.2%) HES-resistant isolates, 4 (4/24, 16.7%) HS-resistant isolates, 1 (1/24, 4.2%) S-resistant isolate, and 10 (10/24, 41.7%) pan-susceptible isolates. 1 (1/27, 3.7%) sample from a retreated patient showed RIF and rifabutin (RFB) resistance classified as RIF mono- or poly- drug resistance TB (RMR/RPR-TB). 9 (9/40, 22.5%) samples showed multi-drug resistance, including 1 (1/13, 7.7%) new patients and 8 (8/27, 29.6%) retreated patients. 6 (6/27, 22.2%) samples from retreated patients showed XDR/pre-XDR-TB. 26 (26/40, 65.0%) pDST results were isoniazid resistance, from 7 (7/13, 53.8%) new patients and 19 (19/27, 70.4%) retreated patients. 11 (11/26, 42.3%) isolates among them were Hr-TB, from 6 (7/13, 46.2%) new patients and 5 (5/27, 18.5%) retreated patients.

#### TB treatment history and treatment outcomes

In 39 retreated cases, 28 (28/39, 71.8%) patients of the R1 group received first-line treatment within two years with treatment failure. 3 patients had multiple interruptions of treatment and 25 patient adhered to taking medication. Most patients came from grassroots hospitals without DST results. 1 patient’s pDST showed RS-TB and his doctors did not adopt the RR-TB result of gDST. 1 patient’s pDST showed RR-TB, she refused to change the first-line medicines. A 75 year old male patient took LFX instead of INH and PZA due to intolerance of adverse drug reactions. 6 (6/39, 15.4%) patients of the R2 group suffered a relapse two years later after completing first-line treatment. 5 (5/39, 12.8%) patients of R3 group failed to respond to the second-line regimen. After RR-TB confirmed by genotypic DST (gDST), the treatment outcome of new patients was better than retreated patients (successful: 20/24, 83.3% VS. 21/39, 53.8%; *P* value = 0.02) (Table 3).

**Table 3:** Treatment outcomes of different types of patients

category	Successful (%)	Unsuccessful (%)			
		LTFU (%)	Died (%)	Failed (%)	Total (%)
**New**	**20 (83.3)**	**4 (16.7)**	**0 (0.0)**	**0 (0.0)**	**4 (16.7)**
FTR	1 (33.3)	2 (66.7)	0 (0.0)	0 (0.0)	2 (66.7)
STR	11 (91.7)	1 (8.3)	0 (0.0)	0 (0.0)	1 (8.3)
LTR	8 (88.9)	1 (11.1)	0 (0.0)	0 (0.0)	1 (11.1)
**Retreatment**	**21 (53.8)**	**14 (35.9)**	**3 (7.7)**	**1 (2.6)**	**18 (46.2)**
FTR	2 (100.0)	0 (0.0)	0 (0.0)	0 (0.0)	0 (0.0)
STR	1 (100.0)	0 (0.0)	0 (0.0)	0 (0.0)	0 (0.0)
LTR	18 (58.1)	11 (35.5)	1 (3.2)	1 (3.2)	13 (41.9)
No treatment	0 (0.0)	3 (60.0)	2 (40.0)	0 (0.0)	5 (100.0)
**Total (N1 = 63)**	**41 (65.1)**	**18 (28.6)**	**3 (4.8)**	**1 (1.6)**	**22 (34.9)**
**pDST: RS-TB**					
Hs	6 (46.2)	6 (46.2)	1 (7.7)	0 (0.0)	7 (53.8)
Hr	9 (81.8)	1 (9.1)	1 (9.1)	0 (0.0)	2 (18.2)
FTR	0 (0.0)	1 (100.0)	0 (0.0)	0 (0.0)	1 (100.0)
STR	5 (83.3)	1 (16.7)	0 (0.0)	0 (0.0)	1 (16.7)
LTR	10 (66.7)	5 (33.3)	0 (0.0)	0 (0.0)	5 (33.3)
No treatment	0 (0.0)	0 (0.0)	2 (100.0)	0 (0.0)	2 (100.0)
**Total (N2 = 24)**	**15 (62.5)**	**7 (29.2)**	**2 (8.3)**	**0 (0.0)**	**9 (37.5)**
**pDST: RR-TB**					
RMR/RPR	1 (100.0)	0 (0.0)	0 (0.0)	0 (0.0)	0 (0.0)
MDR	7 (77.8)	2 (22.2)	0 (0.0)	0 (0.0)	2 (22.2)
XDR/pre-XDR	0 (0.0)	4 (66.7)	1 (16.7)	1 (16.7)	6 (100.0)
LTR	8 (53.3)	5 (33.3)	1 (6.7)	1 (6.7)	7 (46.7)
No treatment	0 (0.0)	1 (100.0)	0 (0.0)	0 (0.0)	1 (100.0)
**Total (N3 = 16)**	**8 (50.0)**	**6 (37.5)**	**1 (6.3)**	**1 (6.3)**	**8 (50.0)**

(1) Treatment outcome: New VS. Retreatment *P* value = 0.02;RS-TB VS. RR-TB *P* value = 0.43. Successful: included cured and Completed treatment; Unsuccessful: included lost to follow-up (LTFU), died, failed, and relapse. So far, there was no relapse case. (2) FTR: first-line treatment regimen; STR: short-term treatment regimen; LTR: long-term treatment regimen. Other abbreviations as Table 1.

#### Phenotypic DST, treatment regimen, and treatment outcomes (table 3)

RS-TB and RR-TB identified by pDST had similar treatment outcomes (successful: 15/24, 62.5% VS. 8/16, 50.0%; *P* value = 0.43). INH-resistant TB (including Hr-TB, MDR-TB, and XDR/pre-XDR-TB) and INH-susceptible TB [including Hs-TB (INH- and RIF-susceptible) and RMR/RPR-TB] had no statistically different treatment outcomes too (successful results: 16/26, 61.5% VS. 7/14, 50.0%; *P* value = 0.48). In the RS-TB group, 81.8% (9 cases including 4 STRs and 5 FTRs) of Hr-TB patients (4 STRs, 6 FTRs, and 1 with no treatment) were treated successfully, while only 46.2% (6 cases including 1 STR and 5 FTRs) Hs-TB patients (1 FTR, 2 STRs, 9 FTRs, and 1 with no treatment) had favorable outcomes. All 6 XDR/pre-XDR-TB patients had poor clinical outcomes. 3/5 (60%) of patients treated with FTR had favorable results, Including one pulmonary tuberculosis patient and two patients with extrapulmonary tuberculosis who underwent surgery (Epididymal tuberculosis and cervical lymph node tuberculosis, respectively). Both STR and LTR for new patients achieved high success rates, 91.7% and 88.9%, respectively. But only 58.1% retreated patients received the LTR successfully. To date, there was no relapse for all patients with successful outcomes.

## Discussion

The Xpert assay is widely used because it not only provides diagnostic results weeks earlier than culture-based methods but also is not vulnerable to other microbial contamination or poor growth. However, the molecular methods cannot determine the proportion of drug-resistant bacteria present in the sample. Thus, molecular methods may be difficult to detect strains with hetero-resistance (mixed wild-type and mutant strains) [21]. Xpert can not identify the mutations outside the RRDR associated with rifampicin resistance in M. tuberculosis, such as V146F and I572F [22]. In some reports, Xpert may generate occasional false-positive RR calls for paucibacillary samples due to delays in the real-time signal generated by assay probes D and E [23]. It can detect silent mutations that do not confer phenotypic drug resistance, such as Q513Q [24], F514F [24–26], K527K [13], et al. The “disputed” mutations, for example, L511P, confer low-level RIF resistance [defined as a minimal inhibitory concentration (MIC) of 0.063-0.5 µg/mL for RIF using the 7H9 Middlebrook medium [27]] and are often classified as susceptible by pDST methods especially the MGIT 960 assay. L511P mutation caused loss of probe A (codons 507–511) signal, while codons 507–510 mutations were rare. It might be the cause for 60.0% of probe A mutant isolates having molecular-phenotypic discordant RIF susceptibility testing results. We proposed to enhance the diagnostic efficiency of the Xpert assay through gene sequencing to identify the mutation sites in discordance RR-TB strains. Furthermore, it is necessary to review the current critical concentration of RIF to ensure that RIF is not applied inappropriately to phenotypic occult RR-TB.

Our study showed that TB treatment history and RR-TB with mono-absent probe A could predict pDST and treatment outcome. Patients infected with molecular-phenotypic discordant RIF susceptibility isolates would likely receive a regimen for drug-susceptible tuberculosis and tended to have poor clinical outcomes. Some doctors believed pDST was more reliable than gDST when their patients were infected with molecular-phenotypic discordant RIF susceptibility isolates, especially when the initial treatment was likely effective. Some grassroots hospitals could not perform gDST tests and had only first-line anti-tuberculosis drugs. It was why some patients had undertaken a first-line regime for a long time without changing their regimens. 28 (28/39, 71.8%) retreated patients in the R1 group received first-line drug treatment with poor results within two years before confirming RR-TB by gDST. It was difficult to conclude either relapse or re-infection without whole-genome sequencing. We observed that RIF resistance increased among retreated patients. 12 (12/13, 92.3%) new local patients in our study were RS-TB by pDST, except the only one imported new case from Singapore was MDR-TB. 15 (15/27, 55.6%) retreated patients were RR-TB by pDST suggested that the TB treatment history might improve the MIC of strains with probe A mutation or lead to other mechanisms of RIF resistance, such as decreased cell wall penetrability to drugs and active efflux pumping [28, 29]. Similarly, Van Deun et al. [30] reported that approximately half of strains from first-recurrence patients with the L511P mutation were RIF-resistant detected by pDST. The high INH-resistant rate was another important reason for the poor clinical outcome of FTR because most of them in our study were actual MDR-TB, and the RS-TB results of pDST led clinicians to make the wrong decision. However, we observed that the successful treatment rate of Hr-TB was higher than Hs-TB in RS-TB group. The INH resistance might contribute to providers’ decision to use expanded regimens. Both STR and LTR for new patients achieved high treatment success rates (91.7% and 88.9%), while only 58.1% of retreated patients who received the LTR were treated successfully due to the 35.5% of patients lost to follow-up. There were 3 patients with chronic hepatitis B in our study cohort, including one new patient and two recurrent patients. They did not interrupt treatment due to abnormal liver function. Nobody suffered from hepatitis C and alcohol abusing. In our STR group, 11 patients used the FQ- and AM/LZD-based regimen, one with the all-oral regimen and one with the 3 LFX-AM-HZE / 9 LFX-HZE regimen. Except for one patient with GFX- and AM-based regimen who was LTFU, the other 12 patients were successfully treated. Such information may be useful for identifying appropriate candidates for STR therapy. Our FQ-resistant patients did not receive STR treatment. There were 7 (7/40,17.5%) FQ-resistant isolates in our cohort, including 1 (1/13, 7.7%) FQ mono-resistance TB from a new patient and 6 (6/27, 22.2%) XDR/pre-XDR-TB from retreatment patients.

Studies suggested that a high-dose RIF (20 mg/kg)-based regimen might be effective for tuberculosis caused by isolates with disputed *rpoB* mutations exhibiting low-level resistance [27, 31, 32]. Furthermore, rifampin doses of up to 20 mg/kg/day produced toxicity comparable to standard doses [33]. However, further studies involving more patients are needed to determine the efficacy of a high-dose RIF-containing regimen for treating cases of disputed *rpoB* mutations with low-level resistance to RIF. Some scholars suggested that not only high-dose RIF but also replacing RIF with RFB could overcome low-level RIF resistance [31, 34]. We found that only 4 (4/40, 10.0%) samples from retreated patients were RFB resistant in our cohort, including 1 (1/4, 25.0%) RMR/RPR-TB isolate, 1 (1/4, 25.0%) MDR-TB isolate, and 2 (2/4, 50.0%) XDR/pre-XDR-TB isolates. More research is needed to prove that RFB offers possibilities for the effective treatment of low-level RIF-resistance TB.

This retrospective study was subject to limitations at the same time. First, we performed Mycobacterium tuberculosis culture using the BACTEC MGIT 960 system, and the pDST by the MYCOTB MIC plate test, rather than the agar proportion method, which might be more sensitive at detecting low-level RIF resistance [35]. Second, this study was a single-center study and relatively small numbers of total specimens. Therefore, the findings here might not apply to a larger population. Third, some authors considered that current critical concentrations of first-line anti-tuberculosis drugs were over-optimistic and should be low to 0.0625 or 0.125 µg/ml, and isolates bearing disputed RIF mutations would be classified as RIF-resistance [36]. Unfortunately, we failed to measure the MIC of strains with disputed *rpoB* mutations in the present study and could only judge them to have low-level RIF resistance based on the discrepant results between the phenotypic and genotypic DST. In addition, a significant limitation of our studies is the lack of genome sequencing to determine the *rpoB* mutation.

In summary, phenotypic susceptibility to RIF in mono-absent probe A strains was common. Many so-called “false-positive” Xpert results were true-positives and that the Xpert assay might be more accurate than initially thought, which reflected insufficient pDST sensitivity. The pDST sometimes missed low-level but probably clinically relevant RIF resistance. Misinterpretation of Xpert results might lead to inappropriate treatment and poor outcomes.

## Conclusions

The Xpert assay is an accurate and time-efficient method and has shown good performance for diagnosing TB and detecting RIF resistance in most settings. However, clinicians must be aware of the limitations of the assay when interpreting the Xpert test results. Our findings described that TB treatment history and RR-TB with mono-absent probe A could predict pDST and treatment outcome, and the disputed mutations implicated poor clinical consequences. Our research results will help clinicians to design reasonable and effective treatment methods in a timely matter.

## Data Availability

The datasets used and/or analysed during the current study available from the corresponding author on reasonable request.

## List of abbreviations

Xpert: GeneXpert MTB/RIF
MTB: Mycobacterium tuberculosis
pDST: phenotypic drug susceptibility testing
RIF/R: rifampicin
TB: tuberculosis
STR: short treatment regimen
DR-TB: Drug-resistant tuberculosis
MDR/RR-TB: multidrug-/rifampicin-resistant tuberculosis
MGIT: Mycobacterial Growth Indicator Tube
FTR: First-line treatment regimen
STR: short-term treatment regimen
LTR: long-term treatment regimen
INH/H: isoniazid
PZA/ Z: pyrazinamide
EMB/E: ethambutol
SM/S: streptomycin
LFX: levofloxacin
GFX: gatifloxacin
MFX: moxifloxacin
AM: amikacin
LZD: linezolid
CFZ: clofazimine
PTO: prothionamide
CS: cycloserine
FQ: fluoroquinolone
BDQ: bedaquoline
WHO: World Health Organization
LTFU: lost to follow-up
XDR-TB: extensively drug-resistant tuberculosis
HIS: hospital information system
BALF: bronchoalveolar lavage fluid
RS-TB: RIF-susceptible tuberculosis
RFB: rifabutin
RMR/RPR-TB: RIF mono- or poly- drug resistance TB
gDST: genotypic DST
MIC: minimal inhibitory concentration
